# Factor-analytic models for genotype × environment type problems and structured covariance matrices

**DOI:** 10.1186/1297-9686-41-21

**Published:** 2009-01-30

**Authors:** Karin Meyer

**Affiliations:** 1 Animal Genetics and Breeding Unit, University of New England, Armidale, NSW 2351, Australia

## Abstract

**Background:**

Analysis of data on genotypes with different expression in different environments is a classic problem in quantitative genetics. A review of models for data with genotype × environment interactions and related problems is given, linking early, analysis of variance based formulations to their modern, mixed model counterparts.

**Results:**

It is shown that models developed for the analysis of multi-environment trials in plant breeding are directly applicable in animal breeding. In particular, the 'additive main effect, multiplicative interaction' models accommodate heterogeneity of variance and are characterised by a factor-analytic covariance structure. While this can be implemented in mixed models by imposing such structure on the genetic covariance matrix in a standard, multi-trait model, an equivalent model is obtained by fitting the common and specific factors genetic separately. Properties of the mixed model equations for alternative implementations of factor-analytic models are discussed, and extensions to structured modelling of covariance matrices for multi-trait, multi-environment scenarios are described.

**Conclusion:**

Factor analytic models provide a natural framework for modelling genotype × environment interaction type problems. Mixed model analyses fitting such models are likely to see increasing use due to the parsimonious description of covariance structures available, the scope for direct interpretation of factors as well as computational advantages.

## Introduction

It has long been recognised that expression of genotypes is altered by environmental conditions. This can result in differences in variability as well as different ranking of genotypes in different environments. Classic analyses of such genotype by environment interaction (G × E) modelled G × E effects in just that manner: as an interaction effect in a two-way classification with genotypes and environments as main effects, in an analysis of variance (ANOVA). Assuming genotypes and interaction effects are random, such basic model generally implies a constant variance of G × E effects and, for more than two environments, a uniform genetic correlation across all environments. Often, this is too restrictive and a number of other models and methods have been developed, both in animal and plant breeding applications; see, for instance, Freeman [1] for a review of early approaches, Cameron [2] for an outline of more modern methods, and James [3] for a recent exposé.

Falconer [4] perceived that treating performance of genotypes in different environments as different, correlated traits provides an alternative way to model G × E effects. As individuals are general limited to a single environment, this relies on the availability of close relatives in the other environments to create genetic links. This approach allows for a more flexible covariance structure which can account for both scale and rank interactions. Generally, the resulting, multi-trait genetic covariance matrix is treated as 'unstructured' which, for *q *environments, comprises *q*(*q *+ 1)/2 distinct covariances. At the other extreme, the 'compound symmetry' structure implied by the two-way ANOVA with interaction involves two parameters, the genetic variance and the variance due to G × E effects. When there are many environments, estimation of an unstructured covariance matrix can be infeasible. Hence, there has been considerable interest in fitting a structure to the covariance matrix which is flexible enough to accommodate heterogeneity of variances and some differences in genetic correlations between environments, but, at the same time, is parsimonious enough to allow estimation of the parameters involved with reasonable accuracy. Recently, interest has focused on structures which utilise the leading principal components of a covariance matrix, as it has become understood that such structures can be fitted directly within the mixed model framework commonly employed for estimation and prediction in quantitative genetic analyses [5,6]. This encompasses both reduced rank and factor-analytic (FA) models. Added impetus for the use of FA models has come from plant breeding applications, especially the analysis of variety trials carried out in a range of locations. There has been increasing use of mixed model methodology in this field, for both the estimation of (co)variance components and the prediction of genetic merit for varietal selection, e.g. [7-11]. This has been stimulated by the recognition that analyses fitting a factor analytic (FA) structure for genotype effects provide the mixed model equivalent to previous, ANOVA based models such as the 'additive main effects, multiplicative interaction' (AMMI) model or regression type models such as the Finlay-Wilkinson model [12]; see Smith *et al*. [13] or Piepho *et al*. [14] for detailed reviews.

A particular G × E problem in livestock improvement is that of international genetic evaluation. For dairy cattle, 'multiple-trait across country evaluation' (MACE) of dairy sires is well established. Loosely described, this utilises a type of adjusted daughter average instead of individual observations, as suggested by Schaeffer [15]. With a considerable number of participating countries, various approaches for a structured parameterisation of the matrices of genetic correlations between countries have been examined, including those fitting reduced rank covariance matrices [16-18] or an approximate FA structure [19,20]. Few other applications have been reported even though maximum likelihood estimation of genetic covariance matrices with a FA structure has been considered early on in other areas [21].

This paper presents a review of FA models and examines their implementation in the standard, linear mixed model framework. Particular focus is on the utility of FA model for genotype × environment type problems, considering scenarios where the genetic covariance matrix is adequately represented by a FA or reduced rank structure.

## The factor-analytic model

### ANOVA based models for G × E problems

A natural formulation for a G × E problem is in form of a two-way classification with interaction. Let *y*_*ijk *_denote the *k*-th record for the *i*-th genotype in the *j*-th environment, *g*_*i *_and *e*_*j *_the additive effects of genotype *i *and environment *j*, *ge*_*ij *_the respective interaction effect, *μ *the overall mean and ϵ_*ijk *_the residual error term. This gives model

(1) *y*_*ijk *_= *μ *+ *g*_*i *_+ *e*_*j *_+ *ge*_*ij *_+ ϵ_*ijk*_

Separation of the interaction component *ge*_*ij *_from the error ϵ_*ijk *_requires repeated records per G × E subclass. Assume we have a 'full' two-way table of G × E effects, i.e. that for *G *genotypes and *E *environments, there are *GE *terms *ge*_*ij*_. This implies that fitting an interaction not only involves a substantial number of additional terms, but can also account for a large proportion of the total degrees of freedom available. Hence, there has been long standing interest in identifying the sources of non-additivity, dating back as far as Tukey [22], and in more parsimonious modelling of the interaction effects.

In addition to reducing the number of effects fitted, structural models can afford an insight into the nature of G × E effects. A bewildering number of alternatives for such models, as used in the analysis of plant breeding trials are catalogued by van Eeuwijk [23,24]. A widely used model, attributed to Finlay and Wilkinson [12], involves a regression on the environmental effect, i.e.

(2) *y*_*ijk *_= *μ *+ *g*_*i *_+ (1 + *β*_*i*_) *e*_*j *_+ ϵ_*ijk*_

with *β*_*i *_the regression coefficient for the *i*-th genotype. The environmental effect *e*_*j *_may be estimated from the data or be comprised of an external, environmental covariable.

A more flexible alternative is a multiplicative model, where each G × E effect is modelled as the product of a genotypic score and an environmental score. More generally, we can model interactions as the weighted sum of products of a number of scores,

(3) $$y_{ijk}=\mu +g_{i}+e_{j}+\sum_{r=1}^{R}\lambda_{r}u_{ri}v_{rj}+\epsilon_{ijk}^{*}$$

with *u*_*ri *_and *v*_*rj *_the *r*-th genetic and environmental score, and *λ*_*r *_the corresponding weight. The number of factors to describe the interaction, *R*, can be at most *G *- 1 or *E *- 1, whichever is the smaller. In practice, *R *is generally chosen much smaller. Parts of the interaction terms not accounted for by the *R *factors fitted are then included in the residual in (3), $\epsilon_{ijk}^{*}$.

A convenient way to determine the scores and weights in (3) is via a singular value decomposition of the matrix formed by the two-way table of G × E effects. This combines the features of ANOVA and factor (or principal component) analysis, and has thus been referred to as FANOVA [25]. Examples of applications, together with discussions on related problems such as tests of significance, partitioning of degrees of freedom, interpretation of factor scores and unbalanced data are given by various authors, e.g. [25-29].

Let **H**, of size *G *× *E *represent the two-way table of G × E effects. Applying a singular value decomposition then yields

(4) **H **= **UΛV'**

with **Λ **= Diag {*λ*_*r*_} the diagonal matrix of eigenvalues, and **U **= {*u*_*ri*_} and **V **= {*v*_*rj*_} the matrices of left and right singular vectors of **H**. **U **is obtained as the matrix of eigenvectors of **HH' **and **V **as that of **H'H**. In the simplest case, the elements of **H **may be estimated as means for individual G × E effects. Other suggestions, in particular for unbalanced scenarios, have been to adjust the G × E cell means for the least-squares estimates of overall mean, genotype and environment effects [25,26].

For such scores, the model given in (3) thus, in essence, describes the interaction terms by considering the *R *leading principal components of **H **only. The resulting model has become known as AMMI model, standing for 'additive main effects, multiplicative interaction' [29,30]. An alternative classification in use is that of a bi-linear or bi-additive model [31]. In some instances, one or both of the main effects are not fitted and the principal component analysis is performed on the combined effects rather than the interaction alone. Some authors refer to such variations of AMMI models as shifted multiplicative models [13,32]. Initial applications of FANOVA or AMMI models considered fixed effects scenarios. Treating environments and interactions as random, Piepho [33] modelled data from plant cultivar trials using the multiplicative models described above, and showed that such models yield a covariance matrix between observations of the same form as that obtained when imposing a factor-analytic structure [34], i.e. given by **ΓΓ**' + **Ψ **with the number of columns of **Γ **equal to the number of factors considered and **Ψ **a diagonal matrix. Smith *et al*. [8] presented a corresponding case with genotypes as random and environments considered to be fixed effects.

### Factor analysis

Loosely speaking, factor analysis is concerned with identifying the common factors which give rise to correlations between variables. This involves fitting a latent variable model. In contrast, principal component analysis aims at identifying factors which explain a maximum amount of variation, and does not imply any underlying model. Let **w **denote a vector of *q *random variables with covariance matrix **Σ**. We then model **w **as

(5) **w **= ***μ ***+ **Γc **+ **s**

with ***μ ***a vector of means, **c**, of length *m*, the vector of common factors, **s**, of length *q*, the vector of residuals or specific effects, and **Γ**, of size *q *× *m*, the so-called matrix of factor loadings. In the most common form of factor analysis, the columns of **Γ **are orthogonal, i.e. ${\gamma^{\prime }}_{i}$*γ*_*j *_= 0 for *i *≠ *j *and *γ*_*i *_the *i*-th column of **Γ**. Hence, the elements of **c **are uncorrelated. Moreover, the common factors are assumed to have unit variance, i.e. Var (**c**) = **I**. Columns *γ*_*i *_are determined as the corresponding eigenvectors of **Σ**, scaled by the square root of the respective eigenvalues. However, **Γ **is not unique and is often subject to an orthogonal transformation to obtain factor loadings which are more interpretable than those derived from the eigenvectors. Finally, the specific effects are assumed to be independently distributed with heterogeneous variances *ψ*_*i*_, and **c **and **s **are assumed to be uncorrelated. This gives covariance matrix of **w **under the FA model

(6) Var (**w**) = **Σ**_*FA *_= **Γ Γ**' + **Ψ**

with **Ψ **= Diag {*ψ*_*i*_} the diagonal matrix of specific variances. This implies that all covariances between the levels of **w **are due to the common factors, while the specific factors account for the additional variance of individual elements of **w**. For *m *common factors, this describes the *q*(*q *+ 1)/2 elements of **Σ**_*FA *_through *p *= *q *+ *mq *- *m*(*m*-1)/2 parameters, consisting of *q *specific variances *ψ*_*i *_and *m*(2*q *- *m *+ 1)/2 elements of **Γ**, with the remaining *m*(*m *- 1)/2 elements of **Γ **determined by the orthogonality constraints. For small *m*, a FA model provides a parsimonious way to model the covariances among a considerable number of variables. As *p *can not exceed the number of parameters in the unstructured case, *q*(*q *+ 1)/2, the number of common factors that can be fitted is restricted. If all specific variances *ψ*_*i *_are non-zero, the minimum number of traits for which imposing a FA structure yields a reduction in the number of parameters is *q *= 4. A FA structure for the variance of **w **is most appropriate if all the *q *traits involved are relatively evenly correlated. In this case, a small number of factors generally suffices to model the covariances among the elements of **w**. The FA model includes many of the commonly employed covariance models for G × E problems as special cases. The simplest scenario is the 'compound symmetry' structure, i.e. Σ = *σ*^2^**11' **+ *ψ***I**, which is a FA model with a single common factor and **Γ **= *σ***1 **(where **1 **denotes a vector with all elements equal to unity) and equal specific variances *ψ *for all variables. Jennrich and Schluchter [34] proposed a FA structure as an option to model the covariances between repeated records, and typical examples where this is appropriate are the 'same' measurements taken in different circumstances, e.g. different time points for longitudinal data, different locations for G × E problems, or different backgrounds in analyses of QTL or gene expression. In contrast to most random regression type 'reaction norm' models which are often invoked for such analyses, the FA approach does not require a continuous 'control' variable and does not imply smooth changes in the trait.

## Mixed model formulation

### Multi-trait model

Consider the linear mixed model

(7) **y **= **X*β ***+ **Zu **+ **e**

with **y **the vector of observations for *q *traits, ***β***, **u **and **e **vectors of fixed effects, random effects and residuals, and **X **and **Z **the design matrices pertaining to ***β ***and **u**. For simplicity, assume **u **represents additive genetic effects only for *N *individuals, with covariance matrix Var (**u**) = **Σ **⊗ **A **and **A **the numerator relationship matrix (NRM). Further, let Var (**e**) = **R**. The corresponding mixed model equations (MME) for a standard, multivariate (MUV) analysis are then

(8) $$\left(\begin{array}{cc} X^{\prime }R^{-1}X & X^{\prime }R^{-1}Z\\ Z^{\prime }R^{-1}X & Z^{\prime }R^{-1}Z+\Sigma^{-1}⊗A^{-1} \end{array}\right)\left(\begin{array}{c} \hat{\beta }\\ \hat{u} \end{array}\right)=\left(\begin{array}{c} X^{\prime }R^{-1}y\\ Z^{\prime }R^{-1}y \end{array}\right)$$

### 'Extended' factor analytic model

The multi-trait framework (8) does not require any assumptions about **Σ **other than that it has full rank *q*. If **Σ **is represented by a FA structure (**Σ **= **ΓΓ**' + **Ψ**), however, an equivalent model to (7) is obtained by fitting the common and specific factors separately [5],

(9) **y **= **X*β ***+ **Z**(**I**_*N *_⊗ **Γ**) **c **+ **Zs **+ **e **= **X*β ***+ Z*c + **Zs **+ **e**

with **c**, of length *mN*, and **s**, of length *qN*, the vectors of common and specific factors, respectively. The corresponding MME are

(10) $$\left(\begin{array}{ccc} X^{\prime }R^{-1}X & X^{\prime }R^{-1}Z^{*} & X^{\prime }R^{-1}Z\\ Z^{{*}^{\prime }}R^{-1}X & Z^{{*}^{\prime }}R^{-1}Z^{*}+I_{m}⊗A^{-1} & Z^{{*}^{\prime }}R^{-1}Z\\ Z^{\prime }R^{-1}X & Z^{\prime }R^{-1}Z^{*} & Z^{\prime }R^{-1}Z+\Psi^{-1}⊗A^{-1} \end{array}\right)\left(\begin{array}{c} \hat{\beta }\\ \hat{c}\\ \hat{s} \end{array}\right)=\left(\begin{array}{c} X^{\prime }R^{-1}y\\ Z^{{*}^{\prime }}R^{-1}y\\ Z^{\prime }R^{-1}y \end{array}\right)$$

Note that Z* is considerably denser than **Z**, containing *m *coefficients *γ*_*ij *_in each row compared to a single element of unity in **Z**. While (10) comprises an additional *mN *equations, the part of the coefficient matrix for random effects is much sparser than for the MUV model, as each element of **A**^-1 ^contributes only *m *+ *q *non-zero elements, compared to *q*^2 ^in (8). With **Ψ **diagonal, $\hat{s}$ can have a number of zero elements if there are 'missing' records: the element for trait *j *and individual *i *is non-zero only if individual *i *or one of its relatives has a record for trait *j*.

In some contexts, the FA model shown in (9) is referred to as 'extended FA' (XFA) model to distinguish it from the equivalent, multivariate model imposing a FA structure on **Σ** (7). For REML estimation of covariance matrices imposing a FA structure, Thompson *et al*. [5] showed that the sparsity of the MME for the XFA model (10) reduced computational requirements dramatically compared to an implementation utilising the standard multi-trait model (8).

### Reduced rank model

A reduced rank model is, in essence, a FA model where specific effects are assumed absent, i.e. **Ψ **= **0**. This is the model proposed by Kirkpatrick and Meyer [6] for parsimonious estimation of genetic covariance matrices. One of the main attractions of the reduced rank model is that it provides a mixed model formulation which allows for genetic covariance matrices that are not of full rank, i.e. it alleviates the need for approximating a reduced rank matrix by a full rank one as required to in the standard MUV implementation (8).

In addition, it can result in computational advantages. Assuming **Σ **can be modelled through the first *m *principal components, the MME have less equations than for the corresponding MUV model. Furthermore, the same arguments for increased sparsity of the coefficient matrix apply as given above for the XFA model. This implies that for *m *= *q*, this parameterisation provides an equivalent model (with **Σ **of full rank) to the standard multi-trait model which not only has a sparser coefficient matrix but also involves random effects which are less correlated. This can reduce both the time per iterate and the number of iterates, in particular in genetic evaluation applications relying on indirect solution schemes. Equally, it may provide some computational advantages for analyses involving a direct solution of the MME.

In the following, we refer to such models as PC models, to describe both reduced (*m *<*q*) and full (*m *= *q*) rank FA models without specific effects.

### Factor rotation

As emphasized above, **Γ **is not unique and, for *m *factors, *m*(*m *- 1)/2 of the *mq *elements are given by orthogonality constraints. Hence, **Γ **is frequently subject to an orthogonal rotation, i.e. we can replace **Γ **by Γ* = **ΓT **for an arbitrary orthogonal matrix **T **without altering the matrix **Σ**_*FA *_modelled, as **ΓΓ**' = Γ*(Γ*)' if **TT' **= **I**. Most commonly, this is done for ease of interpretation – widely used, for instance, in social science applications. However, such transformation can also be utilised to reduce computational requirements, or to provide a parameterisation better suited to variance component estimation.

For *m *= *q *and **Ψ **= **0**, **Γ **is a matrix square root of **Σ**. Let **L **denote the Cholesky factor of **Σ**, i.e. **Σ **= **LL' **with **L **a lower triangular matrix. The Cholesky factor **L **is an alternative matrix square root of **Σ **and, moreover, can be obtained by rotating **Γ**: For **Γ **= **EΛ**^1/2^, with **E **the matrix of eigenvectors of **Σ **and **Λ **the corresponding, diagonal matrix of eigenvalues, it can be shown that **L **= **EΛ**^1/2^**T'**, with **T **the orthogonal matrix of right singular vectors of **L **[[35], p.232]. This implies that we can replace **Γ **in FA models with the *q × m *matrix consisting of the first *m *columns of the Cholesky factor, **L**_*m*_. For variance component estimation, this substitution is useful as the number of non-zero elements of **L**_*m *_is equal to the number of parameters to be estimated, e.g. [8], and as the Cholesky parameterisation is known to improve convergence rates in maximum likelihood estimation.

The triangular nature of **L **can also be advantageous in genetic evaluation, in particular for G × E scenarios where individuals have records in a single location only: As elements above the diagonal are zero, replacing **Γ **with **L**, the rows of Z* are less dense than for a **Γ **with all elements non-zero. Let ${\ell^{\prime }}_{j}$ denote the *j*-th row of **L**. Assuming the Cholesky factorisation has been carried out sequentially, elements *j *+ 1 to *m *of ${\ell^{\prime }}_{j}$ are zero. For an individual with a record in location *j*, vector ${\ell^{\prime }}_{j}$ represents the coefficients in the respective row of the design matrix Z*. If the individual has a record for a single trait (or environment) only, the contribution to Z*'R^-1^Z* is $\sigma_{j}^{-2}\ell_{j}{\ell^{\prime }}_{j}$, with $\sigma_{j}^{2}$ the residual variance pertaining to *j*. It is readily seen that only the block consisting of the first *j *rows and columns of $\ell_{j}{\ell^{\prime }}_{j}$ is non-zero. Hence, the corresponding *m × m *diagonal block in the coefficient matrix corresponding to the common factors **c **has a known sparsity structure, consisting of a dense block, comprising the first *j *rows and columns, and the remaining *m *- *j *rows and columns with all off-diagonal elements equal to zero. For instance, for *j *= 1 there are no off-diagonal elements, for *j *= 2 only the first and second row and column are linked by a non-zero off-diagonal element, and only for *j *= *q *are all *m*^2 ^elements in the diagonal block non-zero. This is readily exploited in both iterative and direct solution schemes. Moreover, for applications with greatly differing numbers of records in different environments, it suggests that numbering environments in decreasing order of the number of records can markedly reduce computational requirements.

### Transforming solutions

As shown, if the genetic covariance matrix is adequately modelled by a FA structure, i.e. **Σ **= **ΓΓ**' + **Ψ**, the standard MUV and the XFA implementation are directly equivalent. In addition, the PC model considering all *q *factors, i.e. decomposing **Σ **= **PP' **(with **P **= **E(Λ **+ **E'ΨE)**^1/2 ^the matrix of scaled eigenvectors of **Σ **or a rotated form thereof), provides a third equivalent model. Hence, solutions for effects in the model can be obtained for one model and are readily transformed to those from another. From (7) and (9),

(11) $$\hat{u}=\{\begin{array}{ll} (I_{N}⊗\Gamma )\hat{c}+\hat{s} & \text{for the XFA model}\\ (I_{N}⊗P)\hat{c} & \text{for the PC model} \end{array}$$

Conversely, as shown by Smith *et al*. [8], we can obtain solutions for the common and specific factors from those in a standard MUV model

(12) $$\begin{array}{ccc} \hat{c}=(I_{N}⊗\Gamma^{\prime }\Sigma^{-1})\hat{u} & \text{and} & \hat{s}=(I_{N}⊗\Psi \Sigma^{-1})\hat{u}. \end{array}$$

Corresponding formulae apply for implementations replacing **Γ **by a rotated matrix Γ* such as **L **and non-equivalent, reduced rank models. Similarly, if estimates of genetic effects for principal components are of interest but a rotated form of **Γ **has been used for ease of computation, these are readily obtained by applying a 'backwards' rotation.

### Example

**Figure 1:**
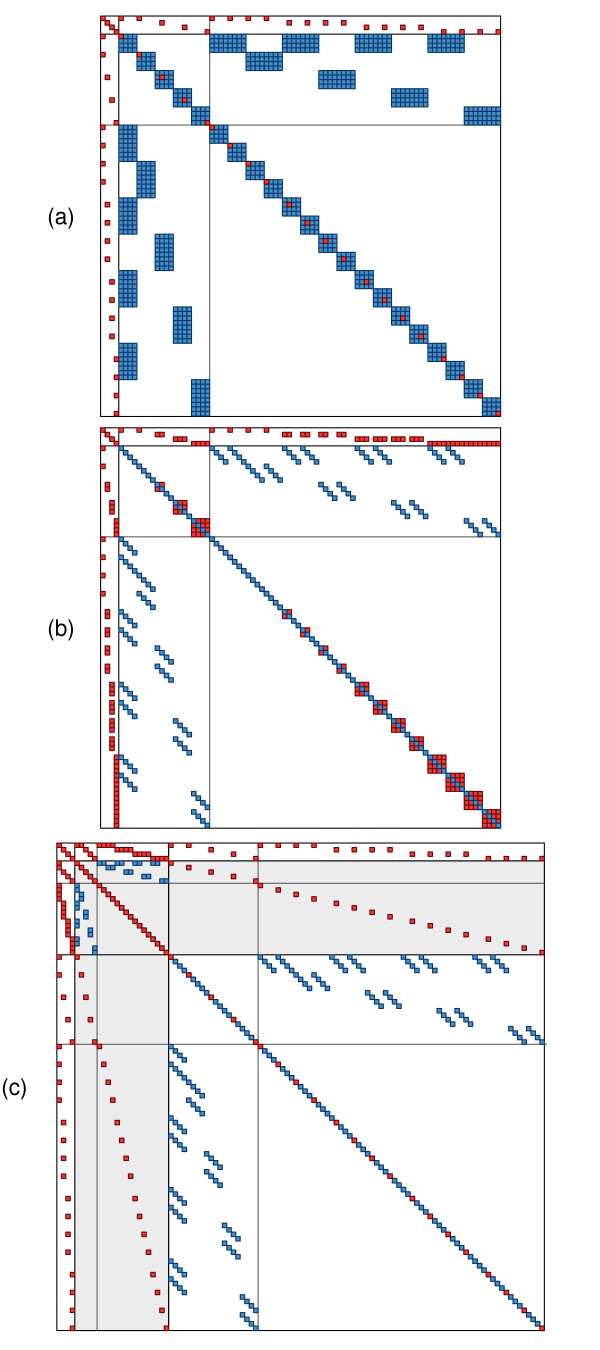
**Fill pattern of coefficient matrix in the mixed model equations for 'toy example' comprising four countries with one global sire used in all countries, one local sire in each country and two offspring per sire; (a) standard multivariate, (b) principal component model fitting all four factors, and (c) extended factor-analytic model fitting one common factor**. (Non-zero elements arising from data part (red square) and from inverse of relationship matrix (blue square))

MUV, PC and XFA models differ greatly in the sparsity of the coefficient matrix in the MME, and the ratio of non-zero off-diagonal elements contributed by the data and the pedigree information. This is illustrated in Figure 1 which shows the fill pattern for a toy example of data for four countries, with two sires used in each country – a global sire and a local sire – and two progeny per sire. This gives a total of 5 sires and 16 progeny and 21 records, assuming we have records on both sires and progeny (with the global sire allocated to country 1). In addition, the MME contain 4 fixed effects, corresponding to the mean in each country. These are represented by the first 4 equations, followed by the equations for the 5 sires and then the 16 progeny, with horizontal and vertical lines separating the blocks for fixed effects, sires and progeny.

For the MUV model, each diagonal block for animals has one element contributed from the data, while each element in the NRM inverse contributes 16 coefficients, resulting in dense diagonal blocks for all animals and a substantial number of off-diagonal elements. This gives 806 non-zero off-diagonal elements or 12.54% filled elements in one triangle (diagonal + off-diagonal) of the symmetric coefficient matrix. The pattern changes substantially when switching to the equivalent PC model fitting all four factors. With factors uncorrelated, each element of the NRM inverse contributes only 4 elements. However, the trade-off is that the design matrix for animal effects is denser, so that there are more contributions from the data part of the MME, i.e. Z*'R^-1^Z*. For an implementation with all elements of **Γ**, the matrix of factor loadings, non-zero this would contribute a dense diagonal block for each animal. However, rotating **Γ **so that elements above the diagonal are zero, this applies only to animals with records in country 4, while the dense blocks for animals with records in other countries are smaller. This is the scenario depicted in part (b) of Figure 1, with 330 non-zero off-diagonal elements in one triangle of the coefficient matrix and a proportion of fill of 6.46%.

Fitting a XFA model, the MME are augmented by the equations for common factors (shown in part (c) of Figure 1 as the part of the equations with a light gray background, again with separation lines between sires and progeny), but sparser yet again. With a single record per individual, there are contributions from the data to only one diagonal element for specific factors, and corresponding off-diagonal elements linking this effect to the corresponding common factors. For this parameterisation, there are 246 non-zero off-diagonal elements and the corresponding fill proportion is 4.20%.

## Multi-trait, multi-environment models

In a more general scenario, we may have multiple traits recorded in each environment. We could then apply the FA decomposition to the complete, multi-trait and multi-environment genetic covariance matrix. This may be necessary if the traits recorded in different locations are quite diverse (but still similar enough to warrant some FA modelling). In other cases, the same traits are of interest in all locations and their covariance matrices may be sufficiently similar across environments that we can utilise the resulting pattern in modelling the joint matrix more parsimoniously.

Most studies on simultaneous modelling of several covariance matrices consider the case of independent groups. Let **Σ**_*ii *_denote the covariance matrix for the *i*-th group. Simple models suggested include proportionality of matrices, i.e. **Σ**_*ii *_= *f*_*i*_**Σ**_11 _(for *i *> 1) with *f*_*i *_the scale factor for group *i*, and the same correlation structure but different variances in different groups, i.e. **Σ**_*ii *_= **S**_*i*_**RS**_*i *_with **S**_*i *_the diagonal matrix of standard deviations for the *i*-th group and **R **the common correlation matrix [36]. Other approaches are based on the spectral decomposition of the matrices. Flury [37] proposed to model similar covariance matrices through common eigenvectors and specific eigenvalues, i.e. **Σ**_*ii *_= **EΛ**_*i*_**E' **with **Λ**_*i *_the matrix of eigenvalues for the *i*-th group and **E **the matrix of common eigenvectors. Later generalisations allowed for partial communality, common subspaces or partial sphericity [38,39] and dependent random vectors [40]. The 'common principal component' approach and resulting hierarchy of models have seen considerable use in the comparison of covariance matrices in evolutionary biology; see Houle *et al*. [41] for a discussion. Pourahmadi *et al*. [42] described a corresponding framework based on the Cholesky decomposition.

Considering traits measured at different stages of development, Klingenberg *et al*. [43] modelled all submatrices of a patterned covariance matrix through common principal components, and emphasized not only that, with rearrangement, this resulted in a block-diagonal covariance matrix of the principal components, but also that further structure (such as reduced rank) could be imposed on this matrix. For *t *traits measured in each of *q *locations, we have a genetic covariance matrix **Σ **with *qt*(*qt *+ 1)/2 distinct elements. A FA structure could be imposed to this matrix as a whole, as described above. For *m *factors, this would involve *m*(2*qt - m *+ 1)/2 + *qt *parameters. Assume in the following that traits are ordered within locations, so that **Σ **has *q*^2 ^submatrices **Σ**_*ij *_of size *t × t *which give the covariances among the *t *traits measured in locations *i *and *j*. It is then conceivable that the covariance pattern among traits across locations is sufficiently similar so that **Σ**_*ij *_= **M**_*i*_**D**_*ij*_**M**_*j*_' with **M**_*i *_the unitary, lower triangular matrix arising from the generalised Cholesky decomposition of Σ_*ii *_(Σ_*ii *_= **M**_*i*_**D**_*ii*_${M^{\prime }}_{i}$ with all diagonal elements of **M**_*i *_equal to unity) and **D**_*ij *_= Diag {$\delta_{k}^{ij}$}. This implies that pre- and post-multiplication of **Σ **with the inverse of **M **= Diag {**M**_*i*_} and its transpose simultaneously diagonalises all *q*^2 ^submatrices **Σ**_*ij*_.

Let **D **= {**D**_*ij *_}, i.e. **Σ **= **MDM'**. **D **is ordered according to traits within environments. It is readily seen that by rearranging the rows and columns of **D **according to environments within traits, we obtain a matrix D* which is block-diagonal with *t *blocks $D_{k}^{*}=\{\delta_{k}^{ij}\}$, of size *q × q*. We can then impose a FA structure on each block in the same way as for the single trait case. Assume $D_{k}^{*}=L_{k}^{*}L_{k}^{{*}^{\prime }}+\Psi_{k}^{*}$, with $L_{k}^{*}$ the matrix consisting of the first *m*_*k *_columns of the Cholesky factor of $D_{k}^{*}$. If we fit a full rank PC model for all $D_{k}^{*}$, i.e *m*_*k *_= *q *and $\Psi_{k}^{*}$ = **0 **(*k *= 1, *t*), and assume all matrices **M**_*i *_are different, **Σ **is described by *p *= *tq*(*t *+ *q *+ 2)/2 parameters. If less factors are considered or matrices **M**_*i *_have some common elements, this is reduced further. For instance, matrices **M**_*i *_may be the same for some environments, or matrices $D_{k}^{*}$ may be proportional to each other.

In certain cases, **Σ **is 'separable', i.e. we are able to decompose **Σ **into the direct product of a *t × t *matrix **Σ**_*T*_, which summarises the covariances between traits, and a *q × q *matrix **Σ**_*Q *_which gives the pattern of correlations between locations and accounts for differences in variability, **Σ **= **Σ**_*Q *_⊗ **Σ**_*T*_. If a FA structure for **Σ**_*Q *_is appropriate, this becomes **Σ **= **Γ**_*Q*_**Γ**'_*Q *_⊗ **Σ**_*T *_+ **Ψ**_*Q *_⊗ **Σ**_*T*_, reducing the number of parameters to describe **Σ **to *p *= (*t*(*t *+ 1) + *m*(2*q - m *+ 1))/2 + *q*, or *p *= (*t*(*t *+ 1) + *m*(2*q - m *+ 1))/2 if **Ψ**_*Q *_= **0**. Smith *et al*. [11] considered such structure in variance component estimation for sugar cane data. Again, there is further scope to reduce the number of parameters if **Σ**_*T *_can be structured as well.

Clearly, being able to impose some common structure on the submatrices of **Σ **can yield a very parsimonious description of the dispersion structure for multi-trait, multi-environment problems, and this is important for variance component estimation. In terms of solving the MME in genetic evaluation, however, differences depend on the solution scheme employed. Say we are considering a FA model using the Cholesky transform, applied to the unstructured *qt × qt *matrix **Σ**, and assume that we are fitting a full rank PC model with *m *= *qt*. We would then have an equivalent linear model (see (9) with Z* = **Z **(**I **⊗ **Q**) and **Q **the Cholesky factor of **Σ**. **Q **is a dense, lower triangular matrix. Hence contributions to the diagonal block of Z*'R^-1^Z* for an animal with records in country *j *would consist of a dense block comprising rows and columns 1 to *jt*. This would be the same if the structure considered above were applicable. However, **Q **would not be dense, but each *t × t *submatrix in the lower triangle would also be a lower triangular matrix. For a solution scheme setting up the MME once and holding them in core, for instance, there would be relatively little advantage of having **Q **with such structure, but for an 'iteration on data' scheme, computational advantages could be substantial.

## Estimation and model selection

Emphasis in this review has been on modelling and prediction, assuming that the genetic covariance matrix has a FA structure. Closely related are the prerequisite tasks of estimation and model selection, i.e. determining how many factors are required. There is substantial body of literature dealing with these topics, and this section is thus restricted to selected pertinent comments.

Most analyses of covariance structures have involved a two-step procedure, first estimating a complete, unstructured covariance matrix and then examining its factors. More recently, direct estimation enforcing a FA structure has been proposed and suitable algorithms for both restricted maximum likelihood (REML) [5,6,44,45] and Bayesian estimation [46] have been described, and mixed model software packages available, such as ASReml [47] or WOMBAT [48], readily accommodate such analyses.

The underlying concept is that only the most important principal components or common factors need to be estimated, while those explaining little variation can be ignored with negligible loss of information. This reduces the number of parameters to be estimated and thus sampling errors. Provided any bias due to the factors that are ignored is relatively small, this is also expected to reduce mean square errors [6].

Furthermore, eliminating unnecessary parameters is likely to make estimation more stable and efficient. For instance, omitting factors with corresponding eigenvalues close to zero reduces problems associated with estimates at the boundary of the parameter space, and can thus improve convergence rates in iterative estimation schemes.

While highly appealing, recent work has identified some unexpected bias in REML estimates of the leading factors in PC models when too few factors are fitted [49]. Briefly, estimation can 'pick up' a wrong subset of factors. Say we fit *m *factors. We would then expect our estimates to reflect the first *m *principal components and any bias in the estimate of **Σ **to be due solely due to factors *m *+ 1 to *q *ignored. However, under certain conditions, one (or more) of the *m *estimated components can represent one (or some) of the lower ranking factors (with smaller eigenvalues) instead. If this is the case, an analysis fitting *m *+ 1 factors typically yields an estimate of the *m*-th eigenvalue which is larger than that from the analysis fitting *m *factors, and the trace of the estimated covariance matrix is increased by more than the value of the additional (*m *+ 1-th) eigenvalue estimated. Another indicator is a large angle between the estimates of the *m*-th eigenvector from the two analyses (the dot product of two normalised vectors gives the cosine of the angle between them): if one of the analyses picked up the wrong direction, this is expected to be orthogonal to the true direction, i.e. we expect it to be close to 90°; see Meyer and Kirkpatrick [49] for details. This inconsistency in estimators implies that we need to choose *m *sufficiently large so that all important factors are included, to ensure that we estimate the leading factors correctly. Paradoxically, this can necessitate the inclusion of some factors with negligible eigenvalues. These can omitted subsequently when using the estimated covariance matrix in a genetic evaluation scheme, i.e. the optimal number of factor to be fitted for estimation and prediction is not necessarily the same. The latter could be determined, for instance, based on selection index calculations and the impact of omitting factors with small eigenvalues on the expected accuracy of evaluation [50].

A number of test criteria to determine the rank of a matrix are available in the literature. Simulation studies examining their utility, however, generally have yielded not very consistent results, both between different tests and in the ability to find the correct dimension (see [49] for references). With mixed model based estimation, model selection based on the log likelihood, information criteria or Bayes factors are an obvious choice. Likelihood ratio tests (LRT) allowing for the fact that testing an eigenvalue for being different from zero involves a one-sided test at the boundary of the parameter space have been described [51,52]. Amemiya and Anderson [53] examined likelihood based goodness-of-fit tests for FA models. Akaike [54] showed that his information criterion (AIC), derived in the context of regression models, was also suitable for FA model selection. However, limited simulation studies in a genetic context have found rank selection based on LRT or AIC to be only moderately successful, with substantial underestimates of the true rank for smaller samples for some constellations of population parameters [49,55]. Future work is needed to examine reliability of model selection for FA models and in more detail.

## Discussion

Mixed model analyses fitting FA models are likely to see increasing use in the future, as higher dimensional analyses considering more than a few traits are becoming more common. This is due to the parsimonious description of covariance structures available, the scope for direct interpretation of factors as well as computational advantages. FA models are most advantageous if all covariances between traits can be attributed to a small number of factors.

Focus in this review has been on modelling of the genetic covariance matrix. Corresponding structures may be applicable for covariance matrices due to other random effects. For scenarios where each individual has records in a single environment only, the residual covariance matrix (**R**) is (block-)diagonal. If there are non-zero residual covariances, we may wish to impose a structure on **R **as well. Simultaneous modelling of several matrices, however, should be carried out judiciously, in particular for variance component estimation: Imposing a structure on the genetic covariance matrix can lead to partitioning of some genetic covariances into the residual part. If the structure imposed on the latter then is too restrictive, problematic estimates for the former may result; see [56] for a cautionary example.

In the context of G × E interactions, separation of genetic effects into common and specific factors is highly appealing, as these factors have an interpretation in their own right. As reviewed above, such models – either ANOVA based or, more recently, employing mixed model methodology – have long been used in the analysis of data from plant breeding trials, and are directly applicable to corresponding problems in animal breeding. For international genetic evaluation, predicted values for common genetic effects of an animal, for instance, could provide global breeding values for that individual. Furthermore, inspection of predictions for the corresponding specific effects could directly reveal its sensitivity to environmental conditions: Similar values for all locations may indicate a good 'all-rounder' while values which are highly variable or are of opposite signs may suggest strong G × E interaction effects.

There has been long standing interest in the use of transformations or reparameterisations of various forms to ease the computational burden imposed by large scale genetic evaluation or variance component estimation problems. Earlier, transformations were mostly applied directly to the data, which limited their applicability. In particular, the so-called canonical transformation was found to be extremely useful for multivariate analyses, as it allowed multivariate analyses to be broken into a series of corresponding, univariate analyses. However, this required equal design matrices for all traits and did not allow for additional random effects; see, for instance, Jensen and Mao [57] for a review. Hence, sophisticated schemes have been developed to augment the data and to extend the range of applications [58,59]. In contrast, FA models involve a reparameterisation of the model, i.e. 'transformations' are applied at the effects level. Thus different design matrices, missing observations or multiple random effects are not an issue. However, the same underlying principles are utilised: computing requirements are reduced by transforming previously correlated effects to be independent and increasing the sparsity of the corresponding MME. Clearly, applicability of FA models depends on the covariance structure among traits or locations being adequately represented by such models. Few studies in animal breeding have addressed this question. Considering genetic correlations for dairy production in 18 countries, Leclerc *et al*. [19] recommended a FA model with 5 common factors, with an average, absolute deviation in genetic correlations from the unstructured case of 0.014. FA models are often been advocated for their parsimony: for problems of relatively high dimensions, reduced sampling variances due to a greatly reduced number of parameters can easily outweigh small biases due to enforcing such structure but, as emphasized above, we need to ensure that the set of factors fitted includes all important factors.

## Conclusion

Factor analytic models, which separate genetic effects into common and specific components, provide a natural framework for modelling G × E interaction and related problems. Moreover, they can substantially reduce computational requirements of mixed model analyses compared to standard multivariate models, both in variance component estimation and genetic evaluation schemes.

## Competing interests

The author declares that they have no competing interests.

## Authors' contributions

All work was carried out by the sole author.
